# Effective Generation of Gynogenic Haploid Zebrafish Embryos Using Low Dosage of UV Rays

**DOI:** 10.19185/matters.201705000003

**Published:** 2017-10-06

**Authors:** Triveni Menon, Janakraj Bhattrai, Sreelaja Nair

**Affiliations:** Department of Biological Sciences, Tata Institute of Fundamental Research

**Keywords:** Gynogenesis, Low Dose UV, Zebrafish

## Abstract

The phenomenon of phenotype manifestation when the single allele in a haploid is affected is desirable for uncovering recessive mutations expeditiously in a diploid organism. However, experimentally generated haploids manifest extensive lethality and a cluster of non-specific developmental defects known as the haploid syndrome. This precludes the use of experimentally generated haploids for genetic screens due to an insufficient number of embryos for screening and the possibility of phenotypes due to the affected gene being masked by the haploid syndrome. We show here that gynogenic haploid zebrafish can be generated by irradiation of spermatozoa with a lower UV dosage than is currently used. This strategy results in reduced haploid lethality, incidence and severity of haploid syndrome. When viewed in the context of zebrafish as a genetically tractable model organism for forward and reverse genetic strategies, these results place zebrafish in a unique niche as a vertebrate in which haploid genetic screens for developmental phenotypes could be successfully attempted.

## Introduction

Forward genetic screens involving chemical mutagens, retroviral and transposon insertions are an unbiased systematic approach towards identification of gene function in several classical animal models. Some crucial factors that researchers have to consider before undertaking phenotype screens in model organisms are the extent of coverage of the genome by the mutagen, cost incurred for the screen, time and labour involved. In general chemical mutagens have a higher mutagenic rate versus insertional mutagens such as retroviruses and transposons [1] [2]. Mutagenesis events occur randomly in the genome introducing heterozygous lesions, which must be homozygosed by generational inbreeding to uncover recessive alleles. The time taken to homozygose mutations in a diploid organism depends on the generation time of the species and whether the phenotype is zygotic or maternal recessive. In case of zebrafish where the average generation time is ~3 months, recessive zygotic phenotypes can be screened earliest at ~9 months and recessive maternal phenotypes at ~12 months from the time of mutagenesis [1] [3]. Considering that the zebrafish genome is ~1.5×10^9^ bp, ~10^6^ haploid genomes will have to be screened for mutations per 1.5 kb of the genome to achieve genome-wide mutagenesis coverage. However, a genetic screen of this magnitude even in zebrafish, the only vertebrate model organism in which saturation genetic screens are a possibility, is daunting even if planned as a coordinated research community endeavor. Since a major factor is the practicality of animal holding space and the animal husbandry cost incurred to house large numbers of filial generations, the time taken from the point of mutagenesis to phenotype screening must be reduced for saturation genetic screens to be a reality.

## Objective

The possibility of phenotype screens in haploids can be a time and cost saving advantage for identification of functions of recessive alleles without the necessity of generating homozygosed diploid embryos. Since there are no naturally occurring haploid vertebrate species, the challenge has been to experimentally generate haploid embryos of diploid model organisms. Haploid vertebrate embryos can be experimentally generated in species from which mature gametes can be extracted, manipulated and in vitro fertilised such as frogs, fish and mammals. In zebrafish destroying either the male or female pronucleus by UV rays can yield gynogenic or androgenic haploid embryos, respectively [4] [5]. However, haploid gynogenic zebrafish embryos generated by current methods have high lethality and manifest a cluster of developmental defects (smaller cell sizes, delayed gastrulation, neural, gut and muscular defects), collectively known as the haploid syndrome making it a suboptimal tool for genetic screens [4]. Our objective was to identify an experimental paradigm that could maximize production of healthier haploid zebrafish embryos, such as haploids manifesting mild haploid syndrome.

## Results & Discussion

### Zebrafish embryos obtained by UV-irradiated spermatozoa are gynogenic haploids

UV-irradiated zebrafish spermatozoa were obtained by exposing spermatozoa to varying durations of UV rays as follows: 30 s (6 millijoules (mJ)/cm^2^), 60 s (12 mJ/cm^2^), 90 s (18 mJ/cm^2^), 120 s (24 mJ/cm^2^) and 150 s (30 mJ/cm^2^). Zebrafish embryos were then generated by in vitro fertilisation (IVF) of mature eggs using either non-UV-treated or UV-treated spermatozoa from different exposures. We refer to the embryos obtained using non-irradiated spermatozoa as control or diploid embryos and those obtained using irradiated spermatozoa as UV-treated or haploid embryos hereafter.

**Figure 1:**
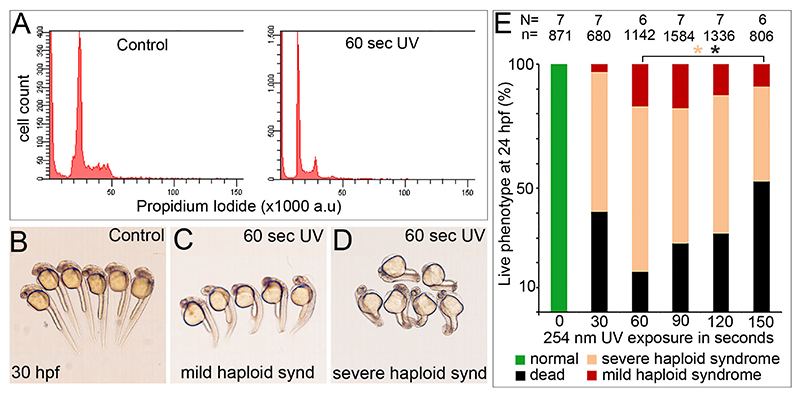
Gynogenic haploid zebrafish generated using spermatozoa exposed to lower UV doses are healthier. **(A)** Flow cytometry analysis of embryos show that the PI fluorescence intensity peaked during G1 phase at ~24 a.u. in control embryos and at ~12 a.u. in UV-treated embryos indicating that genome content in UV-treated embryos is half that of control embryos. **(B-D)** One-day post-fertilisation, control embryos have a well-developed axis, while UV-treated embryos are morphologically different with shorter axis **(C)** and bent tail with cyclopia **(D)** which can be used to categorise embryos into mild and severe haploid syndrome groups. **(E)** Quantification of the phenotype categories at the various UV exposures show that a 60 s exposure elicits minimum lethality and a maximum number of embryos manifesting mild haploid syndrome. Numbers on the top of the histogram represent the total number of experiments (N) and the total number of embryos (n) analysed. * represents p ≤0.05 for severe haploid syndrome (peach asterisk) and lethality (black asterisk) between haploids obtained with 60 s UV exposure when compared individually to 90, 120 and 150 s UV exposures by an unpaired t-test (p <0.05) using Microsoft Excel and GraphPad Prism 5.

We first assayed the DNA content of control embryos and UV-treated embryos by fluorescence-assisted cell cycle profiling at ~24 h post fertilisation (hpf) using Propidium Iodide (PI) staining. As seen in the PI fluorescence intensity plots, control embryos show peak PI fluorescence intensity at ~25 arbitrary units (a.u), while in UV-treated embryos the peak fluorescence decreased by half to ~12 a.u (Fig. 1A and Suppl. Fig. 1A). PI is a double-stranded nucleic acid binding dye and its fluorescence intensity can be correlated with the DNA content of the tissue being analysed. In our experiments, the PI intensity in control embryos is twice that of the PI intensity in UV-treated embryos from each exposure, indicating that the DNA content in control embryos is twice that of UV-treated embryos. Since zebrafish embryos are naturally diploid, our cell cycle profiling data leads us to conclude that control embryos are diploid and the UV-treated embryos are haploids. We performed metaphase chromosome spreads, which allow direct counting of chromosomes to verify the cell cycle profiling data. Control embryos had a chromosome count of 50 while all UV-treated embryos had a chromosome count of 25 (Suppl. Fig. 1B), validating that the UV-treated embryos were haploid.

Mouse and frog haploids harbour smaller sub-nuclei in addition to the zygotic nuclei, which are postulated to be the inert paternal chromatin [6]. Live haploid zebrafish embryos are morphologically indistinguishable from control diploid embryos for the first 10–12 h of development (data not shown). Immunolabelling of control diploid embryos for α-tubulin (microtubules), γ-tubulin (centrosomes) and DAPI (DNA) at 45 min post fertilisation (mpf) revealed cytokinetic microtubular arrays and the dividing zygotic nuclei (Suppl. Fig. 1C). UV-treated haploid embryos from all durations of UV exposures had the cytokinetic microtubular array as was expected. However, UV-treated haploid embryos from the 30 to 90 s exposure had an inactive chromatin streak stretched across the cytokinetic microtubular array. This streak of inactive chromatin was visible as condensed inactive chromatin in UV-treated haploid embryos from the 120 to 150 s exposure. We postulate that the extra chromatin seen in the haploids is the UV irradiated paternal chromatin and we categorize it as cell biologically inactive due to its inability to interact with microtubules and pericentriolar material. A cytokinesis time-course study revealed the presence of the inactive paternal chromatin in all haploids at 60 mpf (data not shown) and we hypothesize that it is eventually extruded from the developing embryo, similar to the polar body chromatin.

### Haploid zebrafish embryos manifest a pleiotropic developmental syndrome known as the haploid syndrome

Live haploids are morphologically indistinguishable from control diploid embryos during the first 20 h of early development (data not shown). By 24 hpf, zebrafish embryos are larvae with a functional circulatory system and well-developed axis with a long tail (Fig. 1B). At ~24–30 hpf, haploid embryos from all UV exposures were morphologically different from control embryos. All haploids could be sorted into two phenotypic categories based on the severity of the haploid syndrome (Fig. 1C, D). The striking feature of embryos with mild haploid syndrome was a shorter body axis and smaller heads. These embryos had mild pericardial edema but were not cyclopic and had no curvature of the tail (Fig. 1C). In comparison, haploid embryos manifesting severe haploid syndrome had cyclopia, severe pericardial edema and a severe bending of the tail in addition to the shortened body axis (Fig. 1D). While control embryos become free-swimming larvae and inflated swim bladders by 4–5 days post fertilisation (dpf), haploid embryos with severe haploid syndrome die by 3 dpf, while those manifesting mild haploid syndrome survive for up to 5–6 dpf, but do not inflate swim bladders.

### Haploid zebrafish embryos generated from shorter UV exposures have higher survival indices and lower incidence of severe haploid syndrome

Since we could reliably categorize haploid embryos based on the severity of the haploid syndrome, we quantified the occurrence of each phenotype category across all UV exposures (Fig. 1E). Control embryos did not manifest any phenotypic abnormalities or lethality. In UV-treated embryos, we observed a trend of decrease in incidence of mild haploid syndrome with increasing duration of UV exposures from 60 to 150 s. Similarly, there was a decrease in the incidence of severe haploid syndrome with increasing duration of UV exposures. However, we found that lethality increased with increasing duration of UV exposures. Since the objective was to identify a UV exposure threshold that would generate healthier haploids, we binned the lethality and severe haploid syndrome as a combinatorial output of the increasing UV exposure. Our data reveal that ~80% of UV-treated embryos from 60 and 90 s exposures and ~90% of UV-treated embryos from the 120 and 150 s exposures either die or manifest severe haploid syndrome. The remaining ~20% embryos from 60 and 90 s exposures and ~10% embryos from the 120 and 150 s exposures manifest the mild haploid syndrome. Strikingly, >95% of embryos from the shortest UV exposure of 30 s either die or manifest severe haploid syndrome. Thus our data show that the clutch of embryos obtained from 60–90 s UV exposures are healthier with significantly lower clutch lethality when compared to the ones generated from 120–150 s exposures. Furthermore, though haploid zebrafish embryos generated from 60 and 90 s UV exposures both yield ~20% embryos with mild haploid syndrome, we conclude that the 60 s exposure is better due to the lower lethality in the clutch in comparison with the 90 s exposure.

The progressive increase in embryonic lethality in gynogenic haploids generated from spermatozoa exposed to UV rays for longer than 60 s suggests that the sperm potentially contributes transcripts or other molecular information for embryonic development in zebrafish. It is unlikely that the degraded paternal DNA is the cause of embryonic lethality as it is not inherited by all cells of the early zygote. Additionally, if the lethality is due to gene dosage errors it would be uniform across all durations of UV exposures as gynogenotes obtained at all UV exposures are haploids. Thus the sperm must contribute information in addition to DNA and centrioles that are potentially progressively degraded with increasing UV exposures, which manifests as UV dose-dependent increase in haploid lethality. It would be interesting to identify such paternal transcripts that the zygote potentially inherits, which are required for normal vertebrate development and survival.

It would be ideal to eliminate the occurrence of the haploid syndrome completely in the gynogenic haploid embryos. A transient heat shock paradigm has been used to induce instant duplication of the genome in zebrafish to generate tetraploids from diploids [7]. It is possible to diploidize haploids by a transient heat shock during the first zygotic mitosis (data not shown) and reduce or eliminate the occurrence of the developmental syndrome completely in such gynogenic diploids, particularly if the haploids are generated using spermatozoa UV-irradiated for 60–90 s.

## Conclusions

Haploid mouse and frogs have to date not been used for genetic screening of developmental phenotypes in whole embryos. This is because of limitations such as implantation failure of haploid embryos in mammals and logistical hurdles in rearing large numbers of mutagenized lines required for saturation genetic screens in both species. This limits the usage of mammalian and frog haploids to the establishment of haploid embryonic stem cells and in vitro experiments [8] [9]. Recently, UV-irradiated carp sperm was shown to be effective at generation of gynogenic haploid zebrafish embryos [10]. Though this cross-species approach generates gynogenic zebrafish haploids effectively, the embryos manifest developmental abnormalities, which are more severe when compared to gynogenic haploids obtained using UV-irradiated zebrafish spermatozoa. This may limit the usage of carp-zebrafish gynogenic haploids for phenotype-driven developmental genetic screens. Zebrafish are free of limitations that prevent use of mammalian model systems for haploid screens and from developmental abnormalities that arise in haploid embryos from cross-species fertilization events, thus proving to be ideal for conducting haploid genetic screens. Our experiments prove that it is possible to generate true gynogenic haploids from inactive spermatozoa that have been UV-irradiated for a short duration of 60–90 s, in contrast to a 120–150 s exposure that is currently practiced. Gynogenic haploids obtained with 60 s UV exposure have a higher survival index with significantly more embryos manifesting mild haploid syndrome, which may be ideal for the use of this genetically tractable vertebrate model organism for haploid developmental genetic screens.

## Limitations

The process of manual egg extrusion which is required for conducting in vitro fertilisation, can deteriorate egg quality in the female (and hence embryo survival). Female fish from which eggs have been extruded must be rested from matings for at least 2 weeks between successive manual egg extrusion procedures.

It is known that androgenic haploids have comparatively severe developmental abnormalities than gynogenic haploids as their production entails UV irradiation of the egg, which degrades RNAs and proteins obligately required for embryonic survival through the first few hours post fertilization when the zygotic genome is transcriptionally quiescent. A complementary optimization strategy to generate healthier androgenic haploid zebrafish might be necessary to minimize egg cytoplasm degradation while optimizing female pronucleus degradation for production of healthier haploid androgenotes. Finally, the potential use of the androgenic haploids and gynogenic haploids generated by UV irradiating spermatozoa for 60–90 s as described herein remains to be tested in a genetic screen for developmental phenotypes. In practical terms, from a single zebrafish male ~800 μl of UV-irradiated sperm solution can be obtained, which can be used to in vitro fertilise ~1500–2000 eggs to obtain gynogenic haploids for genetic screens. For a developmental screen, if the gynogenic haploids are made from a 60 s UV exposure, we expect that 20% (~300–400 embryos) haploids with mild haploid syndrome embryos can be successfully obtained.

Natural parthenogenesis and induced parthenogenesis of the kind described in this study has led to the general view that at fertilization the sperm contributes the paternal pronucleus, centrioles in some species, and not much else that is essential for normal embryonic development. In the last decade, several studies have revealed that the sperm also provides transcripts to the zygote at fertilization which are required for normal embryonic development [11] [12]. In our study, the progressive increase in embryonic lethality in gynogenic haploids generated from spermatozoa exposed to UV rays for longer than 60 s suggests that the paternal genome potentially contributes molecular information essential for embryonic development in zebrafish as well.
